# Evaluating and predicting Klinefelter and Turner syndrome burden in China from 1990 to 2021: A study based on the global burden of disease database

**DOI:** 10.1097/MD.0000000000044519

**Published:** 2025-09-19

**Authors:** Yi Shen, Chuan Song, Yangfan Du, Linlin Wang, Xiaojing Li, Ying Shi, Ye Ding, Song Dong, Hongzhou Liu

**Affiliations:** a Department of Endocrinology, Aerospace Center Hospital, Beijing, China.

**Keywords:** age-standardized rate, China, global burden of disease, joinpoint regression, Klinefelter syndrome, Nordpred age-period cohort, Turner syndrome

## Abstract

Klinefelter syndrome (KS) and Turner syndrome (TS) are among the most common congenital birth defects, with significant differences in both geographic distribution and temporal trends. There is a lack of updated data in China. This study aims to investigate the current situation and trend of the burden of KS and TS 1990 to 2021 in China and to make predictions for the future. This study is a descriptive and epidemiological analysis of the data from the 2021 global burden of diseases, injuries, and risk factors study. The incidence, prevalence, and disability-adjusted life-years (DALYs) of KS and TS in China are assessed by year and age. The estimated annual percentage changes were used to estimate the average changes in age-standardized rates (ASRs). Joinpoint regression analysis was used to assess the trend of KS and TS disease burden from 1990 to 2021. The Nordpred age-period cohort model was used to predict the DALYs, prevalence ASRs, and KS and TS cases from 2022 to 2042. From 1990 to 2021, KS incidence in China decreased by 54%, while TS incidence also showed a decline. However, DALYs and prevalence rates for both syndromes showed variable trends, with KS DALYs increasing and TS DALYs showing significant changes across different age groups. Projections indicate a decline in the number of DALYs and prevalence for both KS and TS from 2022 to 2042, despite an increase in ASRs. The study highlights significant age-related differences in the burden of Chinese KS and TS, with a decreasing trend in KS incidence and an increasing trend in TS-related complications. These findings underscore the need for improved understanding, early detection, and targeted interventions to mitigate the future burden of these diseases.

## 1. Introduction

Klinefelter syndrome (KS) and Turner syndrome (TS) stand as the most prevalent chromosomal anomalies within the spectrum of disorders associated with atypical sex development.^[1,2]^ These conditions are principally characterized by inadequately developed gonads, which yield hypogonadism and infertility when patients reach adulthood. KS is the most frequent cause of male hypogonadism and chromosomal abnormalities. It is found in 0. 2% of the general populace, 4% of patients experiencing male reproductive issues, and an even higher 15% of patients diagnosed with azoospermia.^[3]^ TS is the only viable chromosomal monosomy (45X) in humans, with a prevalence of approximately 1/4000 to 1/2500 in female newborns,^[4]^ and is typically characterized by early signs of loss of ovarian function, but diagnosis is often delayed or missed.^[5–8]^ KS is known as “the forgotten syndrome” and is underdiagnosed because there are no deformities other than a large stature.^[9]^ TS is clinically characterized by short stature and deformity, often detected at 9 to 10 years of age.^[10]^ Despite advances in the genetic and clinical management of KS and TS, the multiple complications and psychological problems that can occur across the life cycle of patients remain severe.^[11,12]^ In addition, previous studies have shown that both KS and TS contribute to a measurable disease burden in terms of disability-adjusted life-years (DALYs) and deaths, particularly due to complications such as cardiovascular disease, metabolic disorders, and increased psychosocial morbidity.^[13,14]^

Studies have shown extensive differences in the temporal trends and geographic distribution of KS and TS worldwide.^[15,16]^ Evaluations of the burden of disease can provide a systematic, scientific, and comprehensive quantification of the health damage caused by diseases, injuries, and risk factors across different regions, ages, times, and genders. One national cohort study estimated that the prevalence of TS showed a gradual increase and that KS was diagnosed at a lower rate.^[6]^ Most studies on KS and TS in China have focused on case reports and single-center data, with research focusing on disease karyotyping and analysis of patients’ basic data.^[17–19]^ There is a lack of studies reporting updated data and estimates of the burden of disease for KS and TS at the national level in China.

Therefore, this study aimed to utilize the 2021 Global Burden of Disease (GBD) Study,^[20]^ to analyze the burden of disease and current status of KS and TS in China from 1990 to 2021, and to predict the future burden of TS and KS disease in China. It would help policymakers assess the burden of KS and TS to inform and guide the development of public health policies.

## 2. Methods

### 2.1. Overview

The GBD 2021 database, coordinated by the Institute for Health Metrics and Evaluation in the United States, leverages a unified methodology to comprehensively analyze the incidence and prevalence of 369 diseases across 204 countries and territories from 1990 to 2021. Detailed descriptions of the background and overview of this study are available in previous studies.^[20]^ GBD data are derived from system evaluations, survey data, hospital administrative data, disease registries, insurance claims, inpatient and outpatient data, and case reports. In the absence of metadata for certain countries or regions, DisMod-MR 2.0 estimates relevant parameters based on data available from neighboring countries. The GBD 2021 database can be accessed through the following website: https://vizhub.healthdata.org/gbd-results/. In this study, we extracted TS and KS data from the GBD 2021 database covering 1990-2021. As the GBD database doesn’t include personal information, informed consent was not required.

### 2.2. Case definition and coding

In the GBD database, KS and TS fall under the category of “Congenital birth defects” within ‘noncommunicable diseases. Disease occurrences were identified according to the International Classification of Diseases (10th edition). Specifically, codes Q96-Q96.9 are defined as TS, and codes Q98-Q98.9 are recognized as KS. Additional details about these specific codes for TS and KS are available on the official GBD website at https://ghdx.healthdata.org/gbd-2021.

### 2.3. Disease burden

This study analyzed the disease burden using 3 principal epidemiological markers: incidence (for children under 5 years of age), prevalence, and disability-adjusted life years (DALYs).^[21]^ Prevalence is expressed as the proportion of all cases that are attributable to a specific disease, whereas incidence denotes the ratio of new cases caused by this disease to the total number of new disease cases. DALYs, accumulated from the total years of life lost due to premature death and disability, represent the proportion attributable to a specific cause out of all causes. When assessing incidence, prevalence, and DALYs, the age-standardized rate (ASR) serves as a critical indicator. ASR effectively minimizes bias caused by differences in age distributions among various regions, and its calculation formula is thoroughly explained in previous studies.^[22]^

### 2.4. Statistical analyses

The burden of disease in KS and TS is expressed as incidence, prevalence, and DALYs by year and age, including numbers and ASRs. estimated annual percentage changes are used to estimate average changes in ASRs.^[23]^ From 1990 to 2021, the average annual percentage change (AAPC) was employed to showcase the time trends for TS and KS in terms of age-standardized incidence (ASIR), prevalence (ASPR), and age-standardized DALYs (ASDR). Age groups, segmented into 5-year intervals, were utilized to illustrate variations and trends across diverse age demographics. Each quantity has been presented and calculated with a 95% uncertainty interval. Joinpoint regression was utilized to calculate AAPCs and corresponding 95% confidence intervals, which were used to assess long-term trends in disease metrics over the study period. Using the age-period-cohort (APC) model, a method commonly utilized in GBD data trend and projection analyses, we analyzed age-standardized DALYs and ASPR trends from 2021 to 2042 for KS and TS.^[24,25]^ All data analysis and visualizations were carried out using R software (https://cloud.r-project.org/, version 4. 2. 2). Statistical significance was set at a 2-sided *P*-value of <.05.

## 3. Results

### 3.1. Overall burden of KS and TS in 1990 and 2021

Table 1 presents the quantity and ASRs of incidence, prevalence, and DALYs for KS and TS in China for the years 1990 and 2021. Between 1990 and 2021, the number of incidence cases of KS in China declined by 54%, whereas the number of prevalence cases and the number of DALYs showed no significant change. During the same period, ASRs in KS showed different trends, with ASIR decreasing by 40%, while ASDR and ASPR increased by 35% and 26%, respectively. TS and KS burdens have similar trends. The same trends were demonstrated for a decrease in the number of incidences (−0.55, 95% CI: −0.57 to −0.0.53) and little change in the other 2 indicators, a decrease in ASIR (−0.46, 95% CI: −0.58 to −0.34) and an increase in ASDR (0.17, 95% CI: 0.11–0.23) and ASPR (0.14, 95% CI: 0.08–0.2).

**Table 1:** The number, ASR, and temporal trend of KS and TS burden in 1990 and 2021 in China.

Burden measures	Number (95% UI)		Annual rate of change	ASRs, per 100,000 (95% UI)		EAPC (95% CI)
	1990	2021		1990	2021	
Klinefelter syndrome						
Prevalence	127,705 (95,980–165,084)	131,686 (99,680–172,229)	0.03 (−0.02 to 0.08)	20.08 (15.08–25.91)	22.14 (16.64–28.83)	0.26 (0.22–0.3)
Incidence	7957 (5605–10,751)	3660 (2618–4975)	−0.54 (−0.56 to 0.52)	1.34 (0.95–1.82)	1.28 (0.92–1.74)	−0.4 (−0.49 to 0.3)
DALYs	795 (381–1652)	830 (394–1751)	0.04 (−0.02 to 0.12)	0.12 (0.06–0.24)	0.13 (0.06–0.27)	0.35 (0.32–0.39)
Turner syndrome						
Prevalence	56,053 (43,586–75,665)	57,262 (45,373–74,376)	0.02 (−0.02 to 0.07)	9.5 (7.38–12.73)	10.07 (8.01–13.21)	0.14 (0.08–0.2)
Incidence	2469 (1835–3341)	1106 (835–1478)	−0.55 (−0.57 to 0.53)	0.48 (0.36–0.65)	0.45 (0.34–0.6)	−0.46 (−0.58 to 0.34)
DALYs	994 (461–1727)	1012 (454–1736)	0.02 (−0.03 to 0.07)	0.16 (0.07–0.29)	0.18 (0.08–0.3)	0.17 (0.11–0.23)

ASR(s) = age-standardized rate(s), CI = confidence interval, DALY(s) = disability-adjusted life year(s), EAPC = estimated annual percentage change, KS = Klinefelter syndrome, TS = Turner syndrome, UI = uncertainty interval.

### 3.2. TS and KS burden in 2021 for different age groups

**Figure 1.**
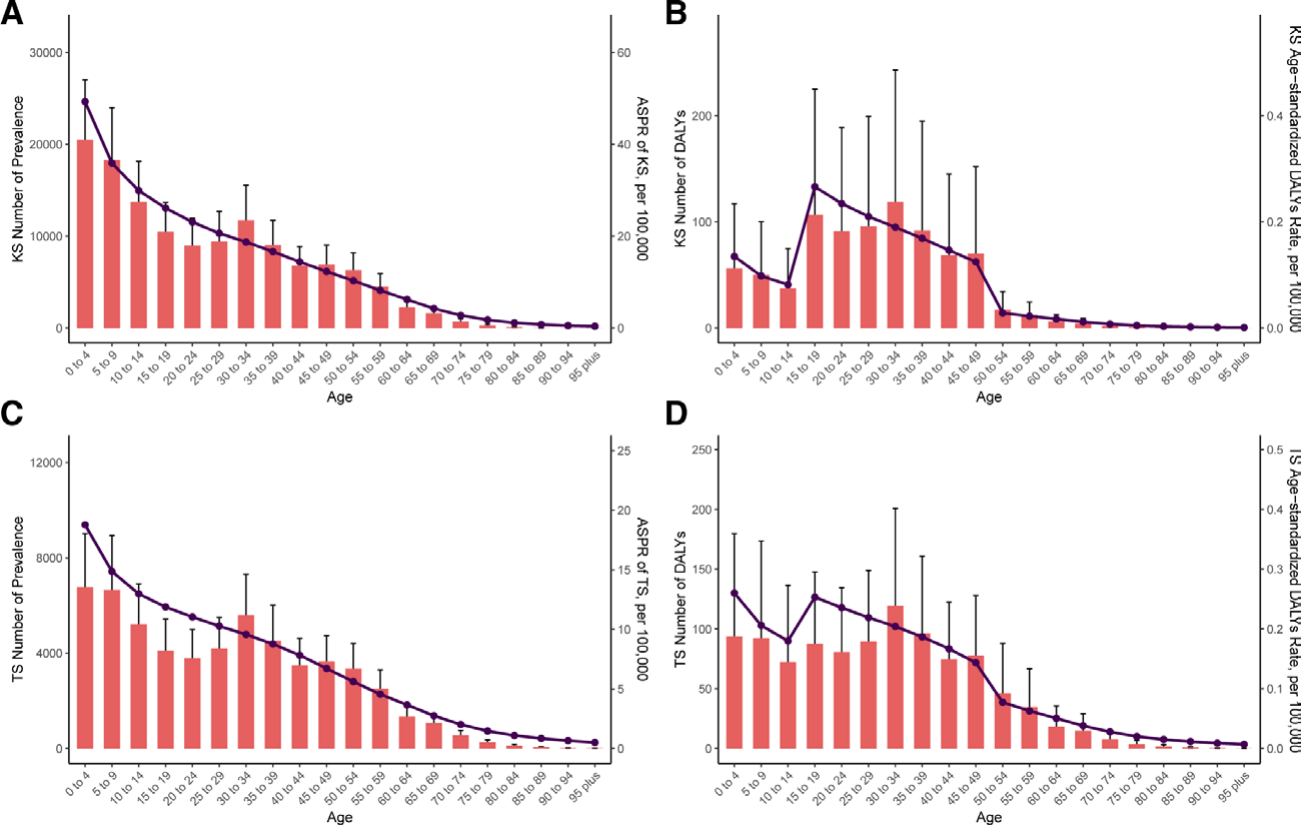
Cases and rates of KS DALYs (A) and prevalence (B) and TS DALYs (C) and prevalence (D) for 2021. Error bars indicate 95% uncertainty intervals as provided by the GBD 2021 database. DALYs = disability-adjusted life-years, GBD = global burden of diseases, KS = Klinefelter syndrome, TS = Turner syndrome.

Figure 1 presents the count and ASRs of KS and TS cases across different age groups in 2021. There is a clear bimodal distribution of KS incidence, with the highest points of the bimodal peaks in the under-5 and 30 to 34 age groups,35 followed by a gradual decline in prevalence with increasing age (Fig. 1A). The trend in age-standardized DALYs echoes this distribution. The number of KS DALYs was predominantly distributed between the ages of 15 to 49 years, and after the age of 50 years, the rate of age-standardized DALYs and the number of DALYs were lower in patients with KS (Fig. 1B). The TS prevalence also showed a bimodal distribution, with the peaks of the bimodal peaks in the <5 and 30 to 34 age groups, and a continuing downward trend in ASPR (highest in the <5 age group) (Fig. 1C). Similar to the trend in DALYs for KS, the DALY rate and number of cases for TS showed a bimodal distribution across age groups. the highest number of DALYs was found in the 30 to 34 year age group, and the highest rate of age-labeled DALYs was found in the <5-year age group (Fig. 1D).

### 3.3. Time trends of age-standardized DALYs, ASPR and ASIR in KS and TS

**Figure 2.**
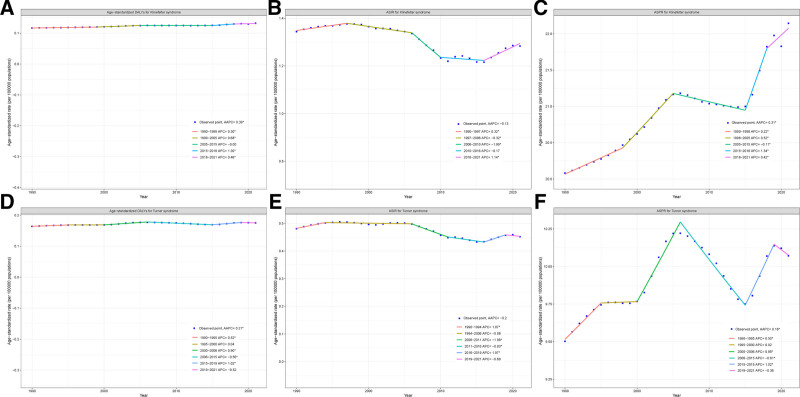
Trends in age-standardized DALYS (A and D), ASIR (B and E), and ASPR (C and F) due to KS and TS from 1990 to 2021 by Joinpoint regression. ASIR = age-standardized incidence rates, ASPR = age-standardized prevalence rates, DALYs = disability-adjusted life-years, KS = Klinefelter syndrome, TS = Turner syndrome.

Figure 2 analyzes the changes of ASDR, ASPR and ASIR of KS and TS in China from 1990 to 2021 by joinpoint regression analysis. From 1990 to 2021, except for ASIR (AAPC = -0.13, *P* >.05), both ASDR and ASPR showed a significant upward trend in KS, China, with AAPCs of 0.39 and 0.31 (*P* <.05), respectively. The ASDR of KS was categorized into 5 APCs, all 4 of which showed a significant upward trend except for the 2005 to 2015 time period (Fig. 2A). Although the AAPC for ASIR in KS was not statistically significant, it showed a significant downward trend in the 1997 to 2006 and 2006 to 2010 timeframes and an upward trend in the 1990 to 1997 and 2016 to 2021 timeframes (Fig. 2B). ASPR can be divided into 5 distinct periods. The APCs for the periods 1990 to 1998, 1998 to 2005, 2015 to 2018, and 2018 to 2021 show a significant upward trend in ASPRs, with only the 2005 to 2015 time period showing a significant downward trend (Fig. 2C). Similar to the trend of disease burden indicators in KS, ASDR and ASPR in TS showed a significant upward trend, with an AAPC of 0.21 and 0.18, respectively, while there was no significant trend in ASIR as a whole (AAPC = −0.21, *P* >.05). The ASDR of TS showed a significant upward trend in the 1990 to 1995, 2000 to 2006 and 2015 to 2019 time periods, while it showed a significant downward trend in 2006 to 2015, with no statistically significant APCs in the other periods (Fig. 2D). While the AAPC of the ASIR for TS was not statistically significant, it showed a significant upward trend in the 1990 to 1994 and 2016 to 2019 timeframes and a significant downward trend in the 2006 to 2011 and 2011 to 2016 timeframes (Fig. 2E). The ASPR for TS shows a complex trend, with significant upward trends in the 1990 to 1995, 2000 to 2006, and 2015 to 2019 time periods, but a significant downward trend in the 2000 to 2006 time period (Fig. 2F).

### 3.4. Projection of age-standardized DALYs and ASPR for KS and TS from 2022 to 2042

**Figure 3.**
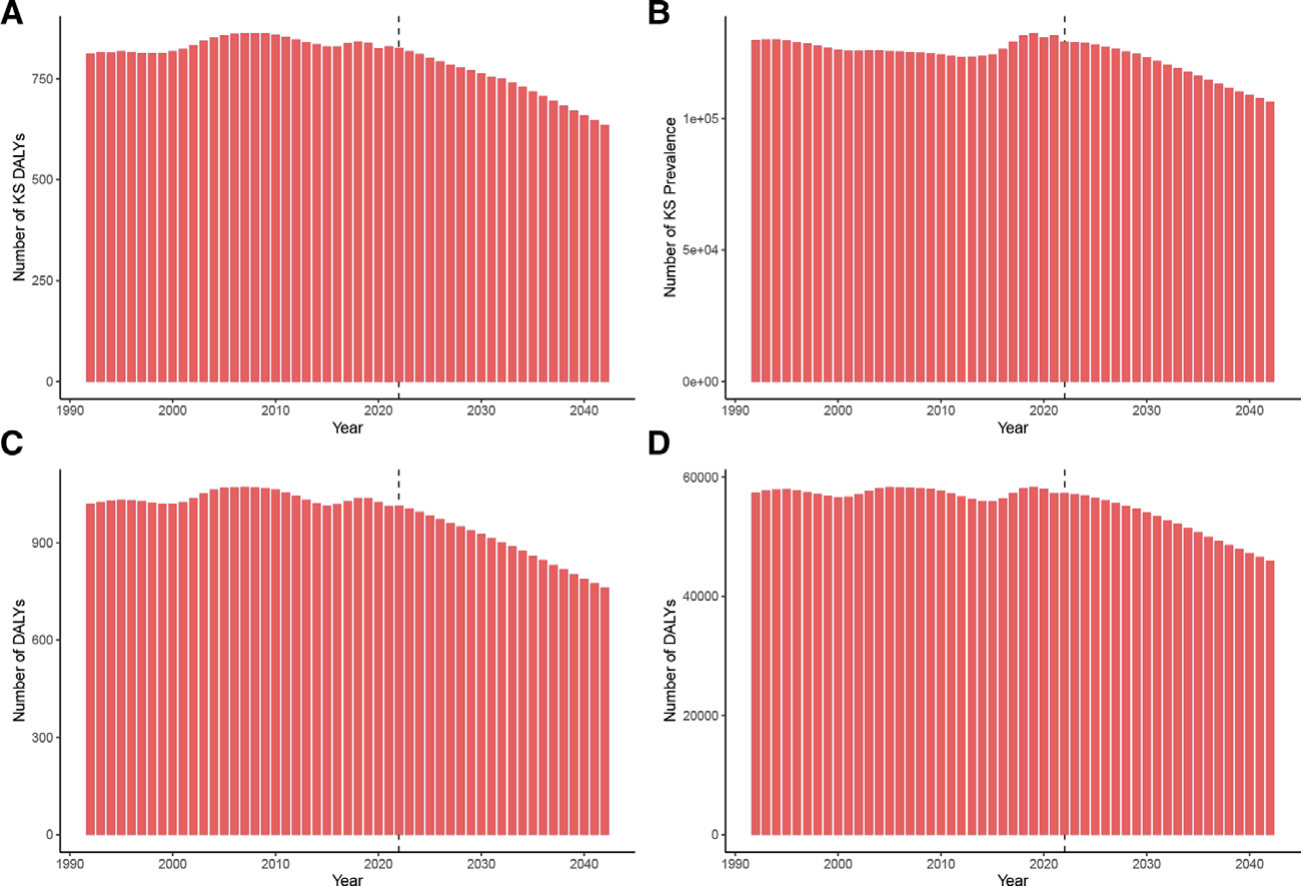
The trends and predictions in numbers of DALYs cases for KS (A) and TS (C), and numbers of prevalent cases for KS (B) and TS (D). DALYs = disability-adjusted life-years, KS = Klinefelter syndrome, TS = Turner syndrome.

**Figure 4.**
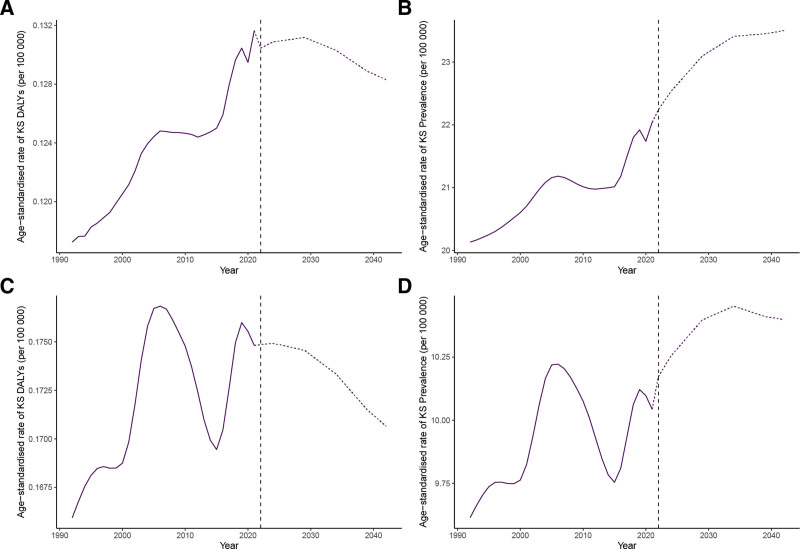
The trends and predictions of age-standardized DALYs for KS (A) and TS (C), and ASPR for KS (B) and TS (D). ASPR = age-standardized prevalence rates, DALYs = disability-adjusted life-years, KS = Klinefelter syndrome, TS = Turner syndrome.

The Nordpred projections depict future trends of the ASDRs and ASPRs for KS and TS diseases over the next 20 years. Both the number of DALYs and the prevalence in KS are projected to show a continuous decline from 2022 to 2042 (Fig. 3A and B). Specifically, the number of DALYs in KS is expected to decrease from 826 in 2022 to 636 in 2042, while the prevalence is projected to drop from 129,255 in 2022 to 106,477 in 2042. Similarly, both metrics for TS also indicate a continuous downward trend (Fig. 3C and D). For TS, the number of DALYs is anticipated to decrease from 831 in 2022 to 614 in 2042, and the prevalence from 129,777 to 106,398 over the same period. Projections of ASDR and ASPR for KS and TS show differing trends. The ASDR for KS is expected to rise briefly before falling from 2022 to 2042, whereas the ASPR will exhibit a continuous upward trend (Fig. 4A and B). In contrast, for TS, the ASDR will show a continuous downward trend, while the ASPR will initially rise and then decline (Fig. 4C and D).

## 4. Discussion

This study provides a comprehensive assessment of the current status and time trends of KS and TS in China from 1990 to 2021, as well as projections for 2022 to 2042 based on GBD 2021 data. We found that both ASRs for KS in 2021 were lower than those in 1990, however, the opposite was true for TS. Both ASRs AAPC for KS showed a significant downward trend, while ASRs AAPC for TS showed a significant upward trend. the Nordpred projections found that from 2022 to 2042, ASRs for both KS and TS showed an upward trend, but the relative number of cases showed a downward trend.

A previous study analyzing neonatal karyotype surveys from the 1960s to 1980s found an upward trend in the prevalence of KS in Western countries.^[26]^ However, our study revealed that the age-standardized prevalence rate (ASPR) of KS in China declined from 1990 to 2021. Variations in the prevalence of KS can theoretically be explained by changes in maternal age, environmentally induced paternal meiotic I errors, and a high willingness to selectively terminate rates of prenatally diagnosed KS.^[27,28]^ Additionally, most epidemiologic studies of sex chromosome aneuploidy are based on data from industrialized countries, and racial differences in the prevalence of KS remain unreported.^[29]^ The reasons mentioned above may also be able to explain the declining trend of KS ASIR in China during the last 30 years.

KS is highly under-diagnosed and diagnosis is often delayed due to mild prepubertal symptoms (median age at diagnosis is 27 years).^[6]^ Only 25% to 35% of men with KS are diagnosed in their lifetime, with most cases going undetected.^[29,30]^ A study in the United Kingdom estimated that of all expected cases, about 10 % were diagnosed prenatally, 6 % in childhood or adolescence, and 19 % in adulthood.^[31]^ Infertility may be most commonly diagnosed in adults because they exhibit symptoms of hypogonadotropic hypogonadism.^[32]^ Single-center study in Denmark found that 20% of KS patients were diagnosed prenatally, and 30% were diagnosed in childhood due to overgrowth and/or behavioral problems.^[33]^ KS is typically diagnosed in adulthood, primarily as part of an infertility evaluation.^[34]^ Our findings are consistent with these previous observations and further highlight the need for improved awareness and early screening in China.

Regarding the burden across age groups, this study found that both the DALY and prevalence burden of KS in China in 2021 were highest among patients under 5 years and those aged 20 to 49 years. International studies also report age- and region-specific variations in diagnosis and prevalence, likely due to differences in healthcare access and awareness.^[35]^ KS is associated with many comorbidities such as malignancy, cardiovascular abnormalities^[36,37]^ and lower life expectancy.^[13,38]^ Early diagnosis and tailored interventions, including language and gonadal function support, are crucial.^[29,33]^ Various strategies to improve diagnosis have been suggested,^[39,40]^ though prenatal diagnosis may increase the risk of adverse outcomes.^[41]^ Testicular examination is a simple yet effective step to improve awareness and early detection^[42,43]^

Although previous studies showed stable incidence from 1970 to 2010.^[44]^ our study observed a decrease in TS incidence in China from 1990 to 2021, which may reflect differences in data modeling, abortion rates, and improved screening programs. This may be due to the models and data used for the GBD database estimation, but it may also be due to the increase in abortions and better screening programs.^[44]^ Population-based epidemiologic studies have estimated the prevalence of TS to be 17 to 88/100,000 women based on the number of women actually diagnosed.^[7,45]^ These differences in prevalence may be due to the fact that standard protocols for chromosomal karyotyping vary quantitatively in the analysis process, as this may lead to the omission of low-level chimeras.^[46]^ The median age at TS diagnosis reported in previous studies ranges from 9.4 to 15 years,^[7,47–49]^ generally younger than for KS, probably due to earlier clinical presentation. Our results align with the known 3 diagnostic peaks – prenatal/perinatal, childhood/adolescence (5–20 years), and adulthood (30–40 years) – and show that the burden of TS in China in 2021 was highest among children under 5 and adults aged 30 to 34, with an elevated burden before age 55. Most TS diagnoses in those aged 5–20 years were due to short stature or delayed puberty, while infertility was the main reason for diagnosis in women aged 20 to 45.^[45,50]^

TS is a multisystem disorder, with an increasing trend in overall mortality,^[44]^ and women with TS are more likely to experience cardiovascular, diabetic, and osteoporotic complications.^[48,51,52]^ Adult patients with TS in the United States had higher rates of obesity, hypertension, and vascular entrapment than European patients.^[53]^ Early diagnosis is critical for the development of relevant treatments and interventions for individuals with TS, such as growth-promoting therapies, age-appropriate puberty induction, prevention of co-morbidities, and cognitive support.^[54,55]^ Increased awareness among healthcare providers, including ENT physicians and primary care, can facilitate earlier TS diagnosis, especially through targeted screening for short stature.^[56]^ Because KS and TS are the same genetic disorders, they cause many similar adverse outcomes as patients grow up. Studies have shown that people with both KS and TS exhibit poor language processing, poor concentration, increased anxiety symptoms, and psychosomatic disorders.^[57,58]^ These reasons result in individuals with KS and TS having unmet social needs and more vulnerable social relationships, and being inferior to their peers of equivalent socioeconomic status in areas such as academic and professional achievement.^[59–61]^

Projections for 2022 to 2042 indicate that the absolute number of KS and TS cases may decrease, while ASRs may rise, possibly due to an aging population structure in China. The treatment and management of KS and TS is a multidisciplinary task, which should include language therapists, endocrinologists, infertility specialists, psychologists, etc, in order to minimize the disease burden caused by KS and TS under the current technological and management conditions as much as possible.^[62,63]^

This study comprehensively evaluated the development status and time trends of KS and TS in China from 1990 to 2021 using the GBD database and made predictions for the next 20 years. However, its limitations should also be noted. First, since GBD data is limited to the national level in China, this study cannot analyze the disease burden at provincial or other levels. Second, the GBD database has not yet collected death-related data for KS and TS, so it is unable to assess their death risk burden. Third, the GBD database does not include individual-level variables such as maternal age or socioeconomic status, which limits our ability to perform stratified analyses by these factors. Finally, many complications occur in patients with KS and TS during their growth process, among which cardiovascular diseases and other comorbidities may affect the assessment of patients’ burden.

## 5. Conclusion

In conclusion, this study reveals significant age-specific differences in the burden of KS and TS in 2021, highlights a declining trend in KS burden and a rising trend in TS burden over the past 3 decades, and provides projections of DALYs, prevalence, and ASRs for both conditions through 2042. Improving awareness, early detection, and timely treatment of KS and TS will be essential to reducing their future burden. These findings offer important evidence to inform the development of targeted public health interventions.

## Author contributions

**Formal analysis:** Xiaojing Li.

**Methodology:** Yangfan Du, Ye Ding.

**Resources:** Yi Shen.

**Software:** Linlin Wang.

**Supervision:** Song Dong, Hongzhou Liu.

**Validation:** Ying Shi.

**Writing – original draft:** Yi Shen.

**Writing – review & editing:** Chuan Song.
